# Reduced Risk of Importing Ebola Virus Disease because of Travel Restrictions in 2014: A Retrospective Epidemiological Modeling Study

**DOI:** 10.1371/journal.pone.0163418

**Published:** 2016-09-22

**Authors:** Shiori Otsuki, Hiroshi Nishiura

**Affiliations:** 1 Graduate School of Medicine, The University of Tokyo, Tokyo, Japan; 2 CREST, Japan Science and Technology Agency, Saitama, Japan; 3 Graduate School of Medicine, Hokkaido University, Sapporo, Japan; China Medical University, TAIWAN

## Abstract

**Background:**

An epidemic of Ebola virus disease (EVD) from 2013–16 posed a serious risk of global spread during its early growth phase. A post-epidemic evaluation of the effectiveness of travel restrictions has yet to be conducted. The present study aimed to estimate the effectiveness of travel restrictions in reducing the risk of importation from mid-August to September, 2014, using a simple hazard-based statistical model.

**Methodology/Principal Findings:**

The hazard rate was modeled as an inverse function of the effective distance, an excellent predictor of disease spread, which was calculated from the airline transportation network. By analyzing datasets of the date of EVD case importation from the 15^th^ of July to the 15^th^ of September 2014, and assuming that the network structure changed from the 8^th^ of August 2014 because of travel restrictions, parameters that characterized the hazard rate were estimated. The absolute risk reduction and relative risk reductions due to travel restrictions were estimated to be less than 1% and about 20%, respectively, for all models tested. Effectiveness estimates among African countries were greater than those for other countries outside Africa.

**Conclusions:**

The travel restrictions were not effective enough to expect the prevention of global spread of Ebola virus disease. It is more efficient to control the spread of disease locally during an early phase of an epidemic than to attempt to control the epidemic at international borders. Capacity building for local containment and coordinated and expedited international cooperation are essential to reduce the risk of global transmission.

## Introduction

An unprecedented major epidemic of Ebola virus disease (EVD) has occurred in West African countries, most notably Guinea, Liberia and Sierra Leone between 2013 and 2016. Since its emergence in late 2013, the epidemic has involved at least 28,599 cases and 11,299 deaths as of the end of 2015 [1]. No licensed vaccines were available before the epidemic and there is no established, specific treatment for EVD, which permitted its initial growth to be nearly exponential [2]. The disease posed a substantial risk of global spread during its early growth phase, and thus, the risk of observing substantial number of deaths. The majority of preventative measures to reduce EVD transmission have been limited to non-pharmaceutical interventions, including case isolation, contact tracing and quarantine, as well as entry and exit screening at borders.

In line with the control and regulation of international travel, International Health Regulations (IHR) have acted as binding international legislation that give the World Health Organization (WHO) legal authority to impose any travel ban or restriction. These regulations also emphasize that any interference with international travel and trade must be minimized [3]. The WHO described the unfolding EVD epidemic as a public health emergency of international concern in August 2014. The declaration aimed to strengthen surveillance and monitoring of the virus on a global scale, including in Guinea, Liberia and Sierra Leone. The WHO has never recommended travel restrictions to delay or prevent the international spread of EVD [4]. Nevertheless, unlike past epidemics of other infectious diseases, and perhaps because of the rapid geographic spread of EVD to cities with international airports, including Nigeria, a substantial number of countries at risk have adopted national policies to respond to the risk of EVD importation at borders. The restrictions ranged from partial cancellation of international flights to full closure of international borders, particularly in countries that belong to the WHO Regional Office for Africa (AFRO). These large scale travel restrictions were implemented based on individual decisions of member countries.

Because of the rapid geographic dissemination of emerging infectious diseases via airline travel, many mathematical modeling studies of global epidemics using airline transportation network data have been reported in the literature [5–10]. A data-assimilation study using epidemic modeling in metapopulation systems enabled researchers to offer real-time forecasting of the geographic spread of EVD [11]. A comparative study of entry and exit screening for EVD emphasized that the cost-effectiveness of entry screening among all incoming travelers at risk is likely to be limited, and argued that exit screening in affected countries would be more beneficial [12]. Another large-scale simulation study offered rapid feedback with respect to the delay effect of the EVD epidemic attributable to travel restrictions, estimating that travel restrictions led to a delay of EVD outbreaks for approximately 30 days in AFRO countries [13]. Moreover, a report issued by the World Bank quantified the economic impact of the 2013–16 EVD epidemic, indicating that there was negative economic growth in affected countries in 2015 [14].

Despite these findings, a post-epidemic evaluation of the effectiveness of travel restrictions using epidemiological data using a simple, tractable method has yet to be reported. Because the major route of EVD transmission is via direct contact or through sexual intercourse [15,16], there may be additional predictors of EVD, other than travel to describe the increased risk of infection in particular individuals, such as social-cultural factors (e.g. a group of people sharing the same language or religion may lead to increased contact). In fact, the importance of accounting for socioeconomic predictors has been highlighted in epidemic modeling studies [17] and should be explicitly tested using empirical data. Employing a simplistic hazard-based regression approach, travel-associated risks and contact behaviors could be modelled in combination with measures of these cultural risk factors. The purpose of the present study is to estimate the effectiveness of travel restrictions, in all countries, that occurred from August to September 2014, using a simple statistical model and exploring additional explanatory variables of the risk of EVD importation.

## Materials and Methods

### Secondary data source

Epidemiological datasets of the date of importation of EVD, as well as the dates and duration of travel restrictions were extracted from publicly available secondary data sources [1,13]. Two authors (SO and HN) validated the date of importation with reference to announcements made by each government. For travel restriction data, a list of countries in which travel restrictions were implemented was collected with the first and last dates of implementation [13].

To capture the airline transportation network-based distance of EVD-free countries from three affected countries in West Africa (Guinea, Liberia and Sierra Leone), network data were obtained from an open source (OpenFlights) [18]. OpenFlights yielded the network data for direct flights between each pair of countries and the total number of direct flight routes was calculated, consisting of 227 nodes (i.e. 227 countries) with 4,598 edges (i.e. 4,598 flights) as of the 10th November 2012.

To explore the potential usefulness of additional explanatory variables, country-specific socio-cultural data were obtained from open access databases [19–23]. Because EVD is transmitted through contact with body fluids, we focused on socio-cultural variables that could potentially mirror physical closeness, including common language (English or French versus others) and religion (Christian or Muslim versus others), the presence of trade, international immigration and policies of exempting entry visas for tourism. English/French and Christian/Muslim were selected because they are widely found in the three countries in this study. Major language was defined as the official language, and major religion was classified as having a coverage of 30% or greater, following analysis of literature in the data source [19]. Furthermore, to compare the effectiveness of travel restrictions by the group of countries, classified by geographic areas, we obtained dataset of regional groups defined by WHO.

### A hazard based model

To determine the risk of transmission on a global airline transportation network in a simplistic manner, the so-called “effective distance”, *D*_eff_, invented by Brockmann and Helbing [24] was employed. Briefly, the effective distance is calculated as the minimum distance between a pair of countries, accounting for the length of path and degree (i.e. number of edges), by employing an adjacent matrix. As the shortest path dominantly predicts the most likely global spread of infectious diseases, the effective distance appeared to be an excellent predictor of arrival time (i.e. the time from emergence to importation of a novel infectious disease) [24]. The validity of predictive performance of the effective distance has been theoretically and empirically argued by Brockmann and Helbing [24] with reference to the global spread of severe acute respiratory syndrome (SARS) and influenza (H1N1-2009). Moreover, the effective distance has been repeatedly used for other application areas including the real time forecasting of the spread of Middle East respiratory syndrome (MERS) and Zika virus [25,26]. Among a total of 227 countries, three affected countries (Guinea, Liberia and Sierra Leone) were grouped into one geographic unit, and subsequently the effective distance from this amalgamated unit to the rest of the 224 countries was calculated.

The hazard function of importation was modeled in two different ways. First, we used the effective distance only, and the hazard of country *i* was modelled as

$$\lambda_{i}=\frac{\beta_{i}}{D_{eff}}$$ (1)

where *β* is a constant (and country-specific). The inverse of effective distance *D*_eff_ is taken, because this formulation allows the median time of importation to be proportional to *D*_eff_, which is consistent with Brockmann and Helbing [24]. Accounting for additional predictors, the second model reads

$$\lambda_{i}=\frac{\beta_{i}}{D_{eff}}\text{exp}\left(\sum_{k=1}^{n}\gamma_{ki}\alpha_{ki}\right)$$ (2)

where *α*_ik_ and *γ*_ik_ are *k*-th explanatory variables and their coefficient of country *i*, respectively, and there are a total of *n* input variables in eq (2).

Under travel restrictions, *β*_i_ and *D*_eff_ in both Eqs (1) and (2) are varied because of the change in the network structure. *β*_i_ was statistically estimated, while *D*_eff_ was manually set in advance of statistical inference. Following an earlier study [13], we assumed that 75% of corresponding flight routes were cancelled during travel restrictions as a default assumption. The arrival time was counted from the 15^th^ of July, 2014 because exponential growth was continuously seen around that time [27–30]. The date on which travel restriction started was set as the 8^th^ of August, 2014 because of the declaration of a public health emergency of international concern. The last date at risk of importation under the travel restriction in the present study was on the 15^th^ of September, 2014 because the effective reproduction number (i.e. the time-dependent number of secondary cases produced by a single primary case) remained above the value of 1.0 by mid-September [28]. The declining phase of the epidemic was excluded from our analysis to permit a simple model using the time-independent hazard function.

A total of 224 countries were divided into three different groups; (i) countries that imported an EVD case before travel restrictions, (ii) countries that imported an EVD cases after travel restrictions, and (iii) countries that did not import any EVD cases. The contribution of group (i) with arrival time *t*_i_ to the likelihood that permits us to estimate unknown parameters, *L*_1_, is

$$L_{1}=\prod_{i}\lambda_{i0}\text{exp}(-\lambda_{i0}t_{i})$$ (3)

where the additional subscript 0 of the hazard *λ* indicates that the effective distance is calculated in the absence of travel restrictions. Similarly, the contribution of group (ii) to the likelihood is given as the product of the probability of escaping from importation for *t*_a_ and that of importing EVD on day *t*_i_-*t*_a_, i.e.,

$$L_{2}=\prod_{i}\lambda_{i1}\text{exp}\left[-\lambda_{i1}(t_{i}-t_{a})\right]\text{exp}(-\lambda_{i0}t_{a})$$ (4)

where *t*_a_ represents the 8^th^ of August 2014, on which the travel restrictions started. Subscript 1 of the hazard *λ* indicates the hazard is calculated using the effective distance under travel restrictions. Lastly, the contribution of group (iii) to the likelihood is calculated as the product of probabilities of escaping for *t*_a_ days before travel restrictions and for *t*_b_-*t*_a_ days after travel restrictions, i.e.,

$$L_{3}=\prod_{i}\text{exp}\left[-\lambda_{i1}(t_{b}-t_{a})\right]\text{exp}(-\lambda_{i0}t_{a})$$ (5)

where *t*_b_ represents the 15^th^ of September, 2014, the last date of our study period. The total likelihood was given by the product *L*_1_*L*_2_*L*_3_ and the maximum likelihood method was employed to infer parameters. The 95% confidence intervals (CI) were derived from the profile likelihood.

### Identifying useful predictors

To identify useful predictors of the risk of importation, we examined the improvement in model fit by adding each single dichotomous variable to two different null models (i.e. a model without any explanatory variables and another model with effective distance only). A likelihood ratio test was used to detect any significant improvement in model fit. Subsequently, a multivariate model was developed. To identify the best fit multivariate model, Akaike’s Information Criterion (AIC) and a backward selection method with the same methodological principle was employed.

### Reductions in absolute and relative risks

Because the best fit model does not necessarily yield the best predictive performance, two different models for the assessment of travel restrictions were used (i.e. a model with effective distance only and the best fit model). Additionally, in countries that imported EVD case(s), there were several intentional importation events for ethical reasons. That is, as part of the evacuation procedure for medical treatment purposes, those working on the ground and who were infected, especially infected physicians and healthcare workers, were admitted to a hospital in their home country. Therefore, we analyzed two different datasets for arrival time; one that included all countries with importation and one that excluded countries that intentionally imported cases for treatment purposes.

Epidemiological assessment of travel restrictions was conducted by calculating the cumulative risk of importation for each country and comparing estimates in two scenarios. Since the hazard is assumed as constant before and after travel restrictions, the cumulative risk of importation is given by one minus the cumulative risk of escaping importation for the period of *t*_a_ and *t*_b_-*t*_a_ days. Namely, the first scenario is the observed scenario in which the risk of importation is calculated as

$$r_{i1}=1-\text{exp}\left[-\lambda_{i0}t_{a}-\lambda_{i1}(t_{b}-t_{a})\right]$$ (6)

for each country *i*. The second scenario is a hypothetical scenario in which no travel restriction takes place.

$$r_{i0}=1-\text{exp}(-\lambda_{i0}t_{b}).$$ (7)

Absolute risk difference because of travel restrictions was then calculated as *r*_i0_-*r*_i1_. The effectiveness was calculated as the relative risk reduction, i.e. 1-*r*_i1_/*r*_i0_. The distribution of these risk reductions were visually investigated as a global map of individual countries and were also examined by WHO region.

### Sensitivity analysis

Sensitivity of the effectiveness of the length of the exponential window was examined. Although the end date of analysis was the 15^th^ of September, 2014 as a default, we also varied the time window from the 12^th^ of September to the 18^th^ of September, 2014. Similarly, sensitivity of the effectiveness of different reductions of travel volume was also examined. Though we assumed a reduction of 75% of flights as a default, we also tested scenarios of 50% and 100% reductions of flights as alternative assumptions.

### Ethical considerations

The present study reanalyzed publicly available WHO data. As such, the datasets used in our study have been deemed as exempt from ethical approval.

### Availability of supporting data

The present study used publicly available data, and essential components of the epidemiological data are downloadable from the WHO website [1].

## Results

### Imported countries

Table 1 shows the list of countries that have experienced importation of EVD in 2014, excluding Guinea, Liberia and Sierra Leone. In total, 12 countries imported EVD case(s). Of these, only three importation events were not associated with medical evacuation. If we limit ourselves to the time from the 15^th^ of July to the 15^th^ of September, 2014, then a total of six countries experienced importation. Of these, four importation events were for medical evacuation purposes.

**Table 1:** The arrival dates of Ebola virus disease by country in 2014.

Country	Date of arrival	Treatment purposes
Nigeria	20/07/2014	No
United States	02/08/2014	Yes
Spain	07/08/2014	Yes
Senegal	20/08/2014	No
United Kingdom	24/08/2014	Yes
Germany	27/08/2014	Yes
France	19/09/2014	Yes
Norway	06/10/2014	Yes
Mali	19/10/2014	No
Switzerland	20/11/2014	Yes
Italy	25/11/2014	Yes
Netherlands	06/12/2014	Yes

The arrival date represents the first date on which an Ebola virus disease infected individual arrived at a country regardless of symptoms upon arrival.

### Identifying predictors

Table 2 shows the result from the likelihood ratio test to identify useful univariate predictor(s) of importation. Compared with null models, the presence of trade and immigration with one of the three affected countries appeared to significantly improve the model fit for the case in which we included a total of six imported countries. When only two countries were included in our analysis, visa exemption was the only variable that improved model fit.

**Table 2:** Improvement in model fit by adding socio-cultural variables.

	Data from 15 Jul to 15 Sep 2014 including importations for treatment purposes (n = 6)		Data from 15 Jul to 15 Sep 2014 excluding importations for treatment purposes (n = 2)	
Variable	Negative log-likelihood	p-value (LR test)^$^	Negative log-likelihood	p-value (LR test)^$^
Constant hazard	52.4	reference	19.7	reference
+Language	52.0	0.777	18.26	0.185
+Religion	51.7	0.459	19.44	0.980
+Trade	47.7	0.005	18.15	0.161
+Visa exemption	52.1	0.943	16.51	0.024
+Immigration	45.8	0.001	17.52	0.075
Constant hazard + *D*_eff_	48.8	reference	18.4	reference
+Language	48.5	0.873	17.04	0.202
+Religion	48.2	0.539	18.18	0.946
+Trade	45.0	0.012	17.16	0.235
+Visa exemption	48.7	0.729	15.56	0.035
+Immigration	43.7	0.003	16.73	0.138

^$^Likelihood ratio test was conducted to judge significant improvement in the goodness of fit compared with baseline models.

*D*_eff_: Effective distance

In the multivariate model eq (2), the use of trade and immigration, in addition to effective distance, appeared to be the best fitting model with minimum AIC value (AIC = 94.1), for a model including importation for treatment purposes (n = 6). This finding that the use of trade and immigration variables improved model fit, agreed with that of univariate analysis. Using the best fit model, the coefficient *β*_i_ was estimated at 0.19 (95% CI: 0.06, 0.32). If we exclude importation for treatment (n = 2), trade and visa exemption were left as explanatory variables in the final model (AIC = 38.3).

### Estimates of effectiveness

**Fig 1:**
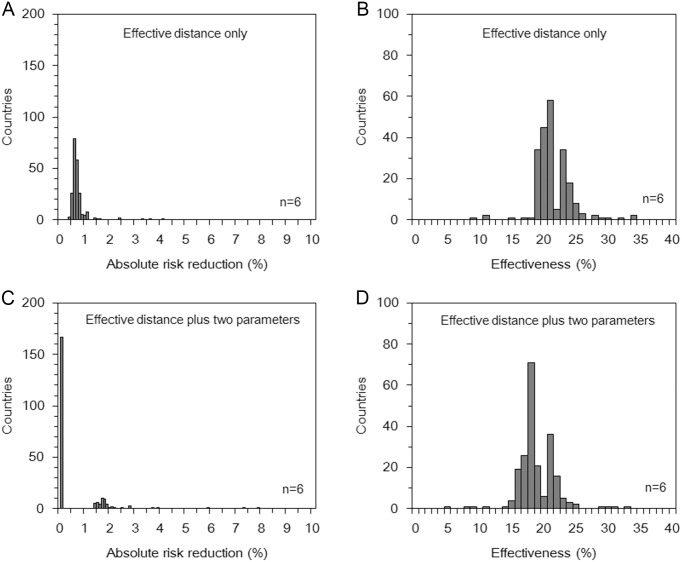
Reduced risk of Ebola virus disease case importation resulting from travel restrictions (all countries with imported cases, n = 6). (A and C) The distribution of absolute risk reduction between scenarios with and without travel restrictions from the 8^th^ of August to the 15^th^ of September, 2014 using (A) the effective distance only and (C) the effective distance and two additional explanatory variables, i.e. trade and immigration. (B and D) The distribution of effectiveness of travel restrictions expressed as the relative risk reduction of importation from the 8^th^ of August to the 15^th^ of September, 2014 using (B) the effective distance only and (D) the effective distance and two additional explanatory variables. These estimates are based on analyses that included countries that accepted importation for treatment purposes.

Fig 1 shows the distribution of estimated effectiveness of travel restrictions across countries. *D*_eff_ at the baseline was 11.5 with the range from 2.3 to 20.0. Although the absolute value of *D*_eff_ is not comparable between different networks, *D*_eff_ when 75% of flights were cancelled was 11.0 with the range from 2.4 to 20.0. Using the effective distance only (Fig 1A), and the best model for analysis of arrival time in all imported countries (n = 6; Fig 1C), the median value of absolute risk reduction was 0.6% (25–75 percentiles: 0.6–0.7) and 0.0% (25–75 percentiles: 0.0–0.1), respectively. Effectiveness, i.e. relative risk reduction, for these models was estimated at 20.1% (25–75 percentiles: 19.5–22.9) and 17.8% (25–75 percentiles: 17.0–20.8), respectively (Fig 1B and 1D). The overall magnitude of estimates and shape of distributions remained similar to those shown in Fig 1, even when we excluded importations for treatment purposes (n = 2; S1 Fig).

### Geographic distribution of effectiveness

**Fig 2:**
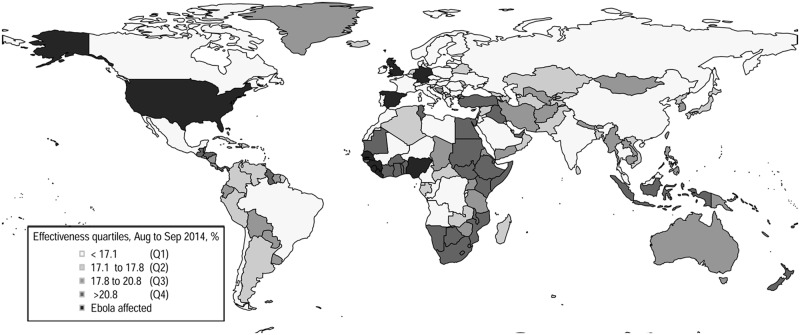
Geographic distribution of the effectiveness of travel restrictions (all countries with imported cases, n = 6). Global distribution of estimated effectiveness of travel restrictions from the 8^th^ of August to the 15^th^ of September, 2014, using the effective distance and two additional explanatory variables (trade and immigration). The estimates are based on analyses that included countries that accepted importation for treatment purposes. The color scale depicts the effectiveness for 227 countries. The first quartile (Q1) represents the countries with the lowest effectiveness, whereas the fourth quartile (Q4) represents those with highest quartile. The nine countries colored black reported an Ebola virus disease case by the 15^th^ of September, 2014; these include Guinea, Liberia, Sierra Leone, Nigeria, USA, Spain, Senegal, the United Kingdom and Germany.

Fig 2 illustrates the geographic distribution of estimated effectiveness (using the best fit model and analyzing all imported countries with n = 6). The highest density was observed on coastal areas of African countries as well as in countries in other parts of the world including the Middle East (Iraq, United Arab Emirates and Turkey), Southeast Asia (Indonesia) and Central and South America (Guyana and Nicaragua). Qualitatively similar patterns were obtained from analysis using the other model and alternative dataset (n = 2).

**Fig 3:**
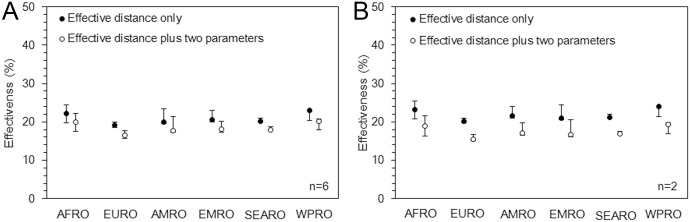
Effectiveness of travel restrictions by World Health Organization (WHO) regional group. Effectiveness of travel restrictions, calculated as the relative risk reduction of importation, by WHO regional group from the 8^th^ of August to the 15^th^ of September, 2014 using the effective distance only and the effective distance and two additional explanatory variables, i.e. (A) trade and immigration and (B) trade and visa exemption. Estimates in panel A are based on analyses that included countries that accepted importation for treatment purposes (n = 6), while panel B excluded the corresponding four countries (n = 2). The black and white dots represent the median distribution within the same WHO region and whiskers extend to lower and upper quartiles. AFRO, EURO, AMRO, EMRO, SEARO and WPRO, respectively, represent regions defined by WHO as Africa, Europe, the Americas, Eastern Mediterranean, South-East Asia and the Western Pacific.

Fig 3 compares the distribution of effectiveness for six WHO regions, using two different predictive models (effective distance only and the best fit model), analyzing all six imported countries. The range of effectiveness was consistently highest in AFRO countries followed by EMRO (Eastern-Mediterranean Region) countries. However, notably, the effectiveness in SEARO (Southeast Asian Region) and WPRO (Western Pacific Region) countries were similarly distributed. Again, the qualitative patterns were similar even when we excluded the four countries that imported cases for treatment purposes.

### Sensitivity analysis

**Fig 4:**
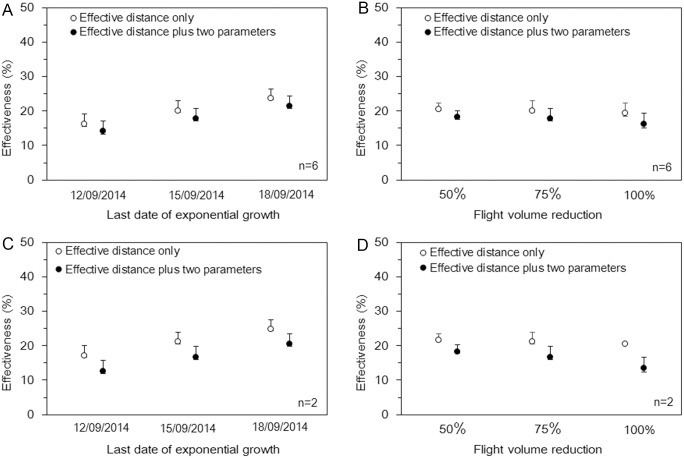
Sensitivity of the effectiveness of travel restrictions to different time windows of the exponential growth period and different volumes of travel reduction. Effectiveness of travel restrictions was calculated as relative risk reduction of importation. (A and C) Effectiveness of travel restrictions by different time windows of exponential growth (i.e. from the 15^th^ of July, 2014 to the 12^th^, 15^th^ and 18^th^ of September, 2014). (B and D) Effectiveness of travel restrictions by different relative reductions of travel volumes (i.e. 50%, 75% and 100%). Estimates in panels A and B are based on analyses that included countries that accepted importation for treatment purposes (n = 6), while panels C and D excluded the corresponding four countries (n = 2). The black and white dots represent the median of effectiveness in countries and whiskers extend to the lower and upper quartiles.

Results from the sensitivity analyses are shown in Fig 4. The estimated effectiveness was slightly elevated when we extended the last date of exponential growth of cases; and this pattern was consistent for different models and datasets. As we decreased the volume of flights, the effectiveness appeared to decrease (given the same empirical data). This effect was more consistently observed in estimates derived from the best fit model than from the model that used the effective distance only.

## Discussion

The present study estimated the effectiveness of travel restrictions for international dissemination of EVD cases, using a hazard-based model and effective distance. In particular, the present study is the first to conveniently use the effective distance in evaluating the effectiveness of travel restrictions using existing empirical data. When we analyzed arrival time data from July to September 2014, during which local preventative measures had yet to be deemed successful, all the models showed that travel restrictions resulted in very small estimates of absolute risk reduction (< 1%) and a relative risk reduction of approximately 20%. The effectiveness of travel restrictions tended to be greatest among AFRO countries, which instituted travel restrictions earliest between August and September 2014. The European countries had the lowest estimated effectiveness, but this is expected because of high connectivity with African countries by air transportation, the acceptance of medical evacuation for international aid workers and their historical context. Full cancellation of flights with affected countries did not increase the overall effectiveness of travel restrictions (Fig 4B and 4D). This could be due to clusters of flights to countries that have not refused international travelers from source countries.

This study highlights that travel restrictions were not as effective as anticipated. As implicated in a large-scale simulation study of travel restrictions [13], an epidemic cannot be prevented with travel restrictions. Further, this very limited effectiveness has been consistently indicated by extensive modeling studies of pandemic preparedness planning [31,32]. Preventative measures at borders may be effective in conjunction with concerted control efforts, including improvement in awareness of the epidemic’s current situation among the public. Rather than expecting border control to be effective, it is better to give priority to locally controlling the spread of disease upon emergence [32,33]. Although the international spread of EVD was limited from 2013–16, our results imply that limited global spread is attributable to successful local control, mainly in the three affected countries, since the effective reproductive number for all three affected countries had fallen to value close to 1 by late September 2014. These effective local interventions included health system strengthening, construction of healthcare facilities, contact tracing efforts and safe burial measures at source [28,34].

Technical improvements in the present study allowed us to obtain our results using a simple model. We have shown that, when a strong correlation exists between the effective distance and arrival time, the effectiveness of travel restrictions can be quantified, even without employing a metapopulation-based epidemic model. Namely, without realizing the epidemic dynamics, the hazard-based model was shown to be useful for evaluating travel restrictions. To our knowledge, the present study is the first to apply the concept of effective distance to evaluate preventative measures against the global spread of EVD. Although the precision of our findings may be less than those of simulation studies reported elsewhere [8,11,13], the present approach captures the key contribution of air travel to the time and risk of importation.

Further analyses to investigate other epidemics and more specific data (i.e., individual-based data), will be essential to fully understand why the presence of trade, immigration and tourist visa exemptions were present in the best fit model. In the case of EVD, the total number of imported countries was very small, and rather than strictly selecting variables with practical (or causal) explanations, it is possible that a few variables that simply statistically improved the goodness-of-fit might have been selected. For this reason, we have also estimated the effectiveness using only the effective distance, throughout the present study. The predictive performance is expected to be improved by selecting truly insightful variables and specific countries particularly at-risk of importation, therefore the present study has shown the potential approaches to exploring epidemiological predictors of importation risk in addition to airline transportation network data.

Our study had some limitations. First, our approach was based on airline transportation network data, and the impact of ground and sea was not taken into consideration. This approach could have underestimated the risk of importation in West African countries, especially in countries that share borders with Guinea, Liberia and Sierra Leone. Second, our network data were imperfect. The dataset was built on direct connecting flights and the degree was determined by the number of flight routes (i.e. not based on the number of passengers). Rather than overcoming these limitations in the network data, greater weight was given to the use of simple open source data [26,35]; however, we believe that using flight route data in our approach was well justified in the context of regression-based modeling using the effective distance. Third, previous studies based on airline transportation network data have used the incorrect assumption that the infected, exporting individual is randomly selected from a source country [36]. Although this assumption was not overcome in the present study, it should be noted that our approach will be able to partially address that assumption by adding key predictive data, such as socioeconomic strata.

In summary, the present study estimated the effectiveness of travel restrictions for EVD importation across the world. The relative risk reduction resulting from travel restrictions was estimated to be approximately 20%; this was not considered a substantial risk reduction to prevent the global epidemic of EVD. Our study highlights the need to strengthen local capacities for disease monitoring and control, rather than relying heavily on border control [37,38]. In addition to local capacity-building and preparedness, we also suggest that rapid local containment should be achieved through coordinated and expedited international cooperation, especially in capacity-limited settings, as was the case with the EVD epidemic [39–41].

## Supporting Information

S1 Fig Reduced risk of Ebola virus disease case importation resulting from travel restrictions (all countries with imported countries, n = 2). (A and C) The distribution of absolute risk reduction between scenarios with and without travel restrictions from the 8^th^ of August to the 15^th^ of September, 2014 using (A) the effective distance only and (C) the effective distance and two additional explanatory variables, i.e. trade and visa exemption. B and D: The distribution of effectiveness of travel restrictions expressed as the relative risk reduction of importation from the 8^th^ of August to the 15^th^ of September, 2014 using (B) the effective distance only and (D) the effective distance and two additional explanatory variables. The estimates are based on analyses that excluded countries that accepted importation for treatment purposes.
(TIF)
