# Supplementary figures and images for: Reduced Risk of Importing Ebola Virus Disease because of Travel Restrictions in 2014: A Retrospective Epidemiological Modeling Study
Source: PLoS One. 2016 Sep 22;11(9):e0163418. doi: 10.1371/journal.pone.0163418 (PMC5033593; doi:10.1371/journal.pone.0163418)

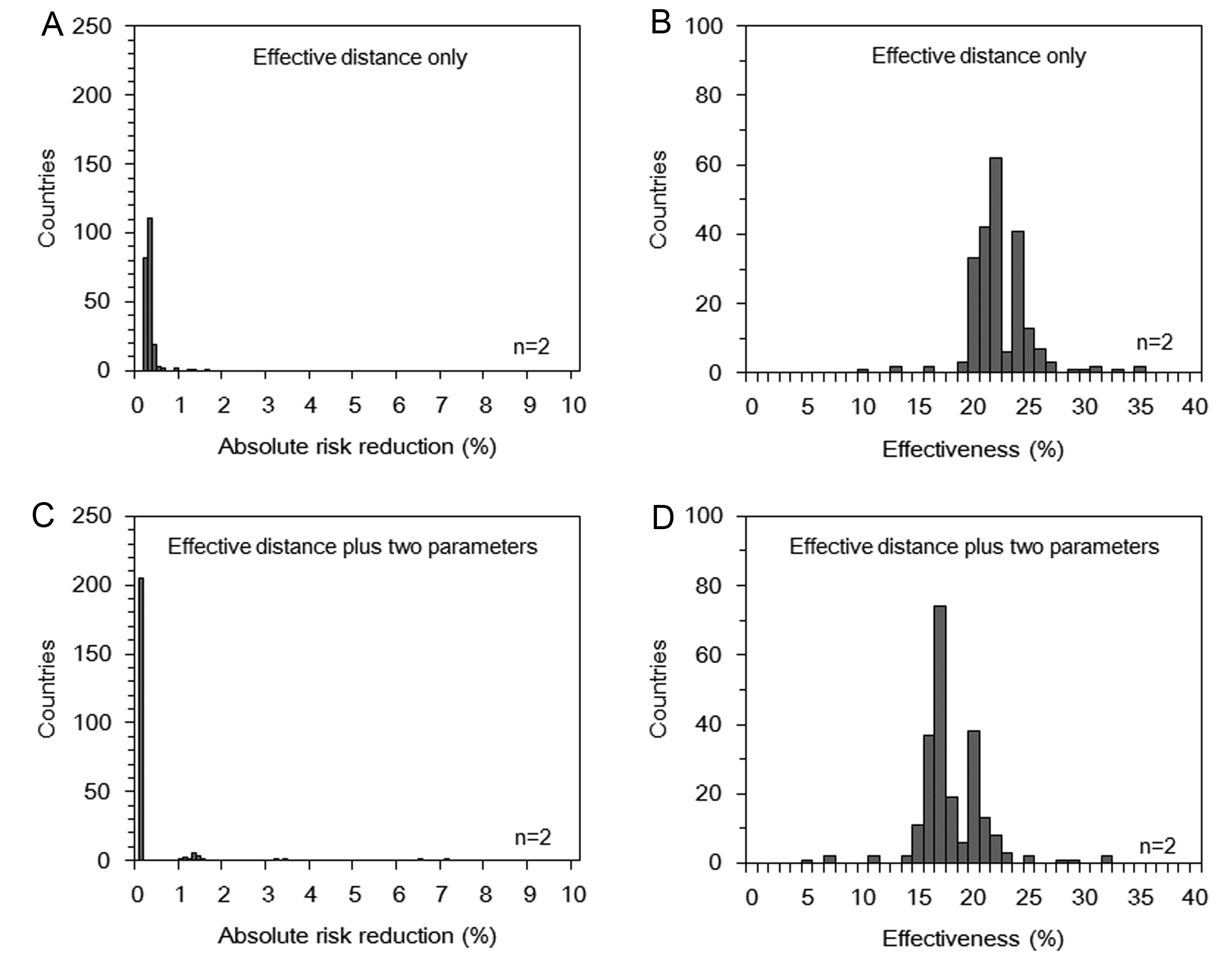

Supplement: S1 Fig — (A and C) The distribution of absolute risk reduction between scenarios with and without travel restrictions from the 8th of August to the 15th of September, 2014 using (A) the effective distance only and (C) the effective distance and two additional explanatory variables, i.e. trade and visa exemption. B and D: The distribution of effectiveness of travel restrictions expressed as the relative risk reduction of importation from the 8th of August to the 15th of September, 2014 using (B) the effective distance only and (D) the effective distance and two additional explanatory variables. The estimates are based on analyses that excluded countries that accepted importation for treatment purposes. (TIF) [file pone.0163418.s001.tif]
